# COVID-19 and Territorial Policy Dynamics in Western Europe: Comparing France, Spain, Italy, Germany, and the United Kingdom

**DOI:** 10.1093/publius/pjab017

**Published:** 2021-06-28

**Authors:** Davide Vampa

## Abstract

This article seeks to assess and explain territorial policy dynamics in five European countries—Italy, Spain, Germany, France and the United Kingdom—from the start of the COVID-19 pandemic up to early 2021. The crisis has clearly highlighted well-known differences between centralized and decentralized systems. Yet focusing on this dichotomy is not sufficient. It is suggested that, while the distribution of authority between central and regional governments matters, policy *dynamics—*that is, how different territorial levels *interact* in policy-making processes—are even more important in driving multi-level responses to the emergency. Whether these dynamics are hierarchical (France), competitive (Italy and Spain), cooperative (Germany) or mixed (the United Kingdom) depends on how pre-crisis institutional, sectoral and political “causal forces” moderate the impact of an exogenous shock.

Between February and March 2020, Europe found itself at the center of a new crisis. After hitting the Chinese province of Hubei, the severe acute respiratory syndrome coronavirus 2 (Sars-CoV-2) causing the coronavirus disease 2019 (COVID-19), rapidly spread into Italy and Spain and then started circulating in all European countries. The epicenter of the pandemic would soon move to the Americas, thus resulting in a truly global crisis, killing hundreds of thousands of people.

Although many observers have focused on the different impact of the pandemic across countries, its “territorial” dimension has only been marginally explored, particularly from a truly comparative perspective. Yet COVID-19 has put “multi-level” governance systems under unprecedented pressure. It has forced public authorities to enforce regional and/or national lockdowns, manage localized clusters of infection, while protecting more demographically vulnerable areas, and coordinate healthcare services across regions in order to avoid overcrowding of hospitals and intensive care units. The question of how, in the face of a crisis, decision-making power should be balanced between national and sub-national governments was not a trivial one and had important social, political and economic implications.

The aim of this article is to assess and explain the territorial policy dynamics triggered by the health crisis in the five largest countries of Western Europe: Italy, Spain, Germany, France and the United Kingdom (UK). Given their size, the issue of which territorial level of government should play a prominent role in the response to the emergency was not straightforward and uncontroversial. In all five countries we observe clear attempts to centralize authority. Yet centralization was achieved in different ways. It was either built with the consensus of sub-national units or (more or less strictly) imposed by national governments. In the latter case, sub-national units could passively accept the leading role of central institutions or could try to resist and oppose it.

Each country followed a different trajectory. Pre-crisis distribution of authority between center and periphery (*structure*) certainly played a role. Yet this factor alone does not explain the emergence of different territorial interactions between center and regions—and among regions. These interactions, or *dynamics*, can be defined by referring to three broad categories: hierarchy, cooperation, and competition. They were shaped by a wide set of pre-crisis “causal forces,” including political actors’ preferences and incentives, the number and characteristics of regional units and the type of health governance.

The article starts by providing a theoretical framework linking crisis and pre-crisis conditions to the emergence of different types of territorial dynamics involving central and regional governments. It then moves to the discussion of the individual case studies in the period from March 2020 to the beginning of 2021. It is shown that, while in France a hierarchical system of crisis management was quickly established, in Germany centralization was achieved by relying on a system of “compensation through-participation,” which allowed to build consensus and coordination between federal government and the *Länder.* In Italy and Spain, after weeks of uncertainty, central governments led the response to the pandemic by limiting the powers of regions. This eventually resulted in increasing central-regional tensions and the emergence of centrifugal political pressures, which are associated with competitive territorial dynamics. Finally, the United Kingdom presents a mixed picture. Initially, a system of cooperation between its four constituent nations seemed to prevail. Yet within England, the absence of strong regional governments, facilitated the creation of a hierarchical system. As the crisis unfolded, however, competitive dynamics emerged among the nations and within England.

The analysis presented here contributes to our understanding of multi-level politics and public policy by highlighting how territorial dynamics, emerging from the interaction between units of government at different territorial levels, shape policy responses to a sudden crisis. This focus on dynamics will in turn help us explain why European countries have not converged to a common model of territorial governance during the first year of the pandemic. Although general attempts at centralization—partly similar to those associated with a military effort—could be detected, they triggered very different types of interactions between sub-national and state-wide authorities. This is not just due to well-known “structural” differences between centralized and decentralized (or unitary vs federal/quasi-federal) countries—all aspects extensively discussed in public and academic debates. The centralized-decentralized dichotomy is too broad and rigid to account for the significant differences existing between countries with equally strong regional and local authorities. It also fails to fully explain inconsistencies observable (at one point in time or over a one-year period) within individual countries. Ultimately, this more nuanced picture can be fully appreciated by considering how a complex set of “pre-crisis” institutional, sectoral and political “causal forces” moderated the impact of an exogenous shock.

## Adjusting to the Shock: Territorial Policy Dynamics

Territorial governance has not been completely absent from public debates focusing on the impact of COVID-19. In fact, many observers have discussed the virtues or failures of centralized and decentralized systems. Often they have done so by referring to just one case or comparing a centralized country to a decentralized one, assuming that each represented two categories of a rigidly dichotomous variable. By looking at five countries with different territorial arrangements and interactions between levels of government, this article aims to provide a more nuanced interpretation of how multi-level systems may respond to a sudden crisis.

A few months after the outbreak, the OECD published a report on the territorial impact of COVID-19 (Allain-Dupré et al. 2020). This is a policy report and, as such, it is more concerned with presenting a list of adopted measures and best practices at different levels of government, rather than building a rigorous analytical framework and testing hypotheses. Yet in the final part of their report, the authors recognize that “the centralisation versus decentralisation debate currently taking place in many countries is a deceptive one” (Allain-Dupré et al. 2020, 54). By reviewing their examples, they come to the conclusion that “a coordinated response by all levels of government, in both federal and unitary systems, can minimise crisis-management failures” (Allain-Dupré et al. 2020, 55). Therefore, implicitly and inductively, the report shows that, rather than just looking at how authority is distributed at different levels, what really matters is how different territorial units *interact*.

To be sure, defining the key differences between centralized and decentralized systems is not a pointless exercise, since it helps to frame an analysis of territorial dynamics. Indeed, before assessing how different levels of government relate to each other—whether, for instance, they compete or cooperate—, it is important to determine how power is formally distributed between them. Here “centralized” and “decentralized” are terms used to define the basic *structural* features of a multi-level system. Decentralization is “a general term for handing down powers to lower levels of government” (Keating 2013, 108). Yet, more precisely, here we refer to a particular type of decentralization, the one which involves meso-level institutions (Scharpe 1993), that is, regional governments. Hooghe, Marks and Schakel (2010) have paid particular attention to this specific level of government and have shown that regional authority has significantly varied across (and within) countries and over time. Of course, decentralization may also involve local (i.e. municipal or departmental) authorities. However, these entities are often “structurally” too small to aspire to compete with higher institutional levels in producing comprehensive policies (Eaton 2017, 34; Vampa 2016, 24). Rather, they often act as agents of central (or regional) governments, particularly in situations of emergency.

Once we have determined how much power regional governments have vis-à-vis the national one, we still do not know much about how the two levels interact and the types of relations existing among different regional units. Do they compete? Do they coordinate? Weaver (2020) has recently provided a comprehensive overview of the variety of policy *dynamics* that may be associated with federal arrangements. The scholar defines policy dynamics as “durable constellations of political actors and causal mechanisms that have distinctive policy consequences over time” (Weaver 2020, 158). They are not only affected by the constitutional status of meso-level authorities, but they are also influenced by additional factors, such as (i) specific characteristics of policy sectors, (ii) the number and characteristics of meso-level units and (iii) incentives for political parties and politicians. These are collectively defined by Weaver (2020, 166) as “causal forces.”

The key policy sector discussed here is, of course, the health sector. Territorial governance of healthcare has clearly played an important role in the relations between central governments and regions. A key characteristic of health systems is the way they are financed. Taxation-based systems have been more subject to territorial pressures and restructuring than insurance-based ones (Costa-Font and Greer 2013, 5–6; Maino and Pavolini 2008). In Italy and Spain, where healthcare is financed out of government tax revenues, regions have been able to gain important policy making autonomy. The same can be said in the case of the United Kingdom, where health services have been devolved. In social insurance systems, like France and Germany, the health finance system and the organization of health care instead tends to be separated from regional governments.

The number and characteristics of meso-level units also varies from country to country and, as argued by Weaver, may shape policy responses in territorial systems. A federation or regionalized system with a large number of meso-level authorities of similar size will face more coordinating challenges than a system with a small number of units or one that is clearly dominant (i.e. it is significantly larger or economically more powerful than the others). Ultimately, however, it is politics and politicians that make decisions and drive territorial interactions. Indeed, as highlighted by Keating (2013, 89–90), decentralization itself does not determine whether *centrifugal* or *centripetal* politics will prevail. The existence of a territorially fragmented parties and party systems, strong regionalist and sub-state nationalist parties may result in centrifugal politics, which, in turn, favors competitive and uncoordinated policy dynamics and responses.

COVID-19 can now be added to the picture. The pre-crisis “causal forces” described above interact with the “exogenous shock” associated with the pandemic. This results in a specific set of policy dynamics, which are not necessarily “durable,” since they might be contingent upon the immediate health emergency. Yet an unexpected and unprecedented crisis like the coronavirus pandemic may also become a “critical juncture,” placing institutional arrangements on a path that is then very difficult to alter (Capoccia and Keleman 2007). This is particularly true if the state of emergency or disruption continues over a significant period of time. Therefore, what we observe in year following the pandemic may be indicative of a new “equilibrium” in territorial relations within multi-level systems.

**Figure 1:**
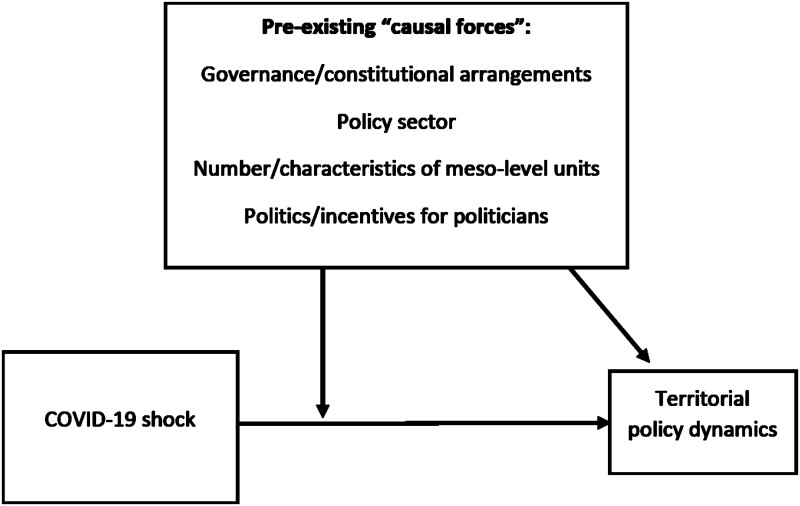
Territorial policy dynamics as the result of the interaction between the COVID-19 “exogenous shock” and pre-crisis “causal forces.”

Generally, the framework adopted by this article looks at the mechanisms that link the outbreak to the emergence of specific policy dynamics. As shown in Figure 1, the impact of the pandemic on policy dynamics is “moderated” or “absorbed” by the “causal forces” described by Weaver, which already affected policy dynamics before the crisis (this explains the two arrows starting from the “causal forces” box).

Yet, apart from providing a general definition of what they are, so far we have not discussed what forms policy dynamics can take. After reviewing the literature on federalism, Weaver (2020, 159) outlines twelve different policy dynamics, which “affect the direction of policy change within a federal system and the heterogeneity of policy choices, outputs, and outcomes across states and provinces.” To be sure, they describe very well the way national and sub-national actors interact in a wide range of contexts. Weaver’s typology helps categorize different policy paths leading to the elaboration and implementation of specific policies. At the same time, however, his categories seem to be mainly applicable to “ordinary” interactions emerging domestically without any significant “exogenous” interference. In order to study multi-level (short- to medium-term) policy responses to COVID-19, it might be more useful to refer to more general “macro-categories” of cooperation/coordination, competition and hierarchy, which, ultimately, subsume Weaver’s more detailed classification of policy dynamics.

Literature focusing on domestic responses to external challenges has assessed the different impact of cooperative or competitive dynamics and centralizing (hierarchical) pressures on policy outcomes. For instance, in her study on multi-level adaptation to Europeanization, Börzel (2001) has shown how an external process like European integration might redistribute resources from regions to the central state and trigger cooperative or competitive strategies depending on the formal and informal characteristics of domestic institutions (the latter can be assimilated to Weaver’s “causal forces”). Of course, a sudden health (and then socio-economic) crisis like the one caused by the coronavirus pandemic is very different from the construction and development of a supranational political system like the European Union. Yet categories similar to Börzel’s can be used in our analysis of the territorial impact of COVID-19. Indeed, also the pandemic facilitated a sudden redistribution of powers to central governments, which were in a more advantageous position than regional governments in providing an immediate response to the crisis. Hence, central governments could impose a more “hierarchical” system of governance to face the emergency, whereby regions and other sub-national units would just become agents of centrally planned strategies. This is also what occurred after 2008 in the aftermath of the financial crisis and Great Recession, when central governments imposed austerity measures to sub-national administrations and limited their spending autonomy (Toubeau and Vampa 2020). Of course, a hierarchical system in which central institutions play a clearly dominant role can be more easily established in countries where governance arrangements and incentives for politicians already led to centralization before the crisis. In more decentralized countries, instead, centralizing pressures may face some resistance and favor the emergence of competitive or cooperative strategies adopted by domestic actors, including regional governments (Börzel 2001, 34).

The next section uses the categories defined above to show how five European countries have responded to the emergency. In all cases, we see clear attempts to centralize power which, however, result in different policy dynamics.

## Different Territorial Responses to COVID-19

### France: Reinforcing “dirigisme”

Among the countries considered here, France is the only clearly “unitary” one, that is, it does not rely on federal or quasi-federal institutional structures, which grant significant (formal or informal) authority to sub-national government. Yet it has been included in this analysis for two reasons. First, being very close to the “ideal-type” of centralized country, it can be used as a sort of yardstick to detect and assess attempts at centralization occurring in the other four cases—and check whether a general convergence to stronger central government has occurred in Western Europe. Secondly, since regional authorities do exist in France, it would be interesting to see if COVID-19 has provided them with new opportunities to strengthen their role and shift the balance of power between center and periphery to their advantage.

Immediately after it became clear that the coronavirus was circulating in Italy, neighbouring France triggered a response plan to the pandemic called ORSAN (*Organisation de la réponse du système de santé en situations sanitaires exceptionnelles*). Created in 2014, this plan organizes, *for the whole territory*, the response of the health system in exceptional health situations. It was partially applied in 2014 for the arrival of the Ebola virus in France, in 2015 for seasonal influenza, and for the terrorist attacks in Paris in November 2015 and Nice in July 2016 (Duguet and Rial-Sebbag 2020, 176). The plan adopted and deployed centrally by the Health Ministry in 2020 was an ORSAN REB, a component of ORSAN focusing on epidemiologic and biologic risk (*risque épidémique et biologique*).

The guidelines of the ORSAN plan clearly state that, in situations of crisis, a two-level functional “hierarchization” of healthcare facilities is established (Ministère de Solidarités et de la Santé 2019, 24). This allows “concentration of treatment” in pivotal first-line establishments while, at the same time, ensuring that town ambulatory medicine and other health care facilities continue to function as usual (Barro et al. 2020, 19). Hierarchy and concentration thus were the key principles of the French response to the pandemic.

Within this framework, the regional level is subordinated to the national one: the main function of the former is to implement the policies elaborated by the latter (Ministère de Solidarités et de la Santé 2019, 189). It is true that in 2010 the French government decided to create Regional Health Agencies (*Agences Régionales de Santé*, ARS). Yet this development did not result in an empowerment of French regions. Rather, it allowed the central state to play a greater role than before (state representatives are included in the ARS supervisory board). As argued by Jones (2013, 225), the room for autonomy and innovation of regional agencies is so minimal, while vertical oversight is so strong, that the reform leading to the creation of ARS significantly increased central control over health policymaking. This is well summarized by Simonet (2013, 264), pointing out that

[i]n practice, the central government runs the ARS: regional health policies are not independent from national health policy; the ARS has no discretionary use of funding (such as for hospital construction or renovation) and must comply with a nationally—rather than regionally—defined health expenditures target.

These pre-crisis policy sector arrangements are also consistent with the general weakness of French regions, which, following a “productivist” ideal, were reduced from 24 to16 in 2014, continued to carry little weight compared to municipalities and departments, and remained very dependent on central government (Brennetot 2018, 181). Additionally, the impact of the pandemic on policy dynamics was affected by incentives for politicians, who, in France, have traditionally seen the sub-national dimension as subordinated to national politics. This is well exemplified by the phenomenon of “*cumul des mandats*” whereby French politicians combine local and national offices (Knapp 1991). Although the *cumul* has been limited by law (Durovic 2019, 1488), the Prime Minister Édouard Philippe was re-elected mayor of the city of Le Havre soon after the peak of the first wave, also thanks to his growing popularity during the national health emergency (Mallet 2020). Philippe and the President of France, Emmanuel Macron, played a key role in the management of the pandemic. Although a sort of “dualism” emerged between them—Philippe would eventually be replaced by a new Prime Minister in July—they did not face significant competition or opposition from subnational authorities. Consequently, they could just rely on a hierarchical system of governance without the need to reach consensus or establish coordination mechanisms with territorial actors.

In fact, the pandemic just reinforced the centralized and hierarchical nature of territorial policy dynamics in France (Kuhlmann et al. 2021). The government quickly adopted a decree-based national strategy underpinned by a “wartime rhetoric” (Yan et al. 2020, 767). The main decisions were taken by the “Defence council,” led by the French president and including the Prime Minister and the ministers discretionarily selected by the President (Hassenteufel 2020, 174). Following a “*dirigiste*” logic, patients on life-support machines were transferred from more to less affected regions (*The Economist* 2020; Onishi and Méheut 2020)—often using high speed trains.

France’s administrative structure also enabled it to centrally enforce containment in specific areas of the country before a national lockdown was imposed. In early March, the French government’s strategy was localized, focusing on “*clusters*” and, later, “*zone de circulation active du virus*,” where cases of infections were concentrated. Yet this “territorial” strategy was implemented and monitored by following the guidelines of the Health Ministry under the close supervision of the *préfets*, representatives appointed by central government at the departmental level—an example of this is the case of Brittany’s department of Morbihan, which experienced early diffusion of coronavirus (Croguennec 2020).

As highlighted by Hassenteufel (2020, 174), the *préfets* and the ARS opposed a number of local initiatives, including local decrees introducing a curfew or the compulsory wearing of masks. ARSs, directly linked to the national health ministry, were primarily involved in local steering, while local and regional authorities, lacking competencies in the area of healthcare were largely excluded. In early April municipal governments in some cities and towns instituted mandates for wearing masks in public, which, however, were soon blocked by central government (Büthe et al. 2020). Only in July did the French government issued a national order which made wearing masks obligatory in public. Testing initially proved to be a challenge for the French hierarchical model, which, compared to decentralized Germany—but also Italy and Spain—, struggled to deploy an extensive network of testing services (Onishi and Méheut 2020). Thus, almost two months after the beginning of the outbreak, France—together with the United Kingdom—was well behind the other three more decentralized countries in the level of testing and caught up only after the first wave of the pandemic had subsided.

Generally, the French case shows that in an already centralized system an external shock may lead to even more centralization and the establishment of a hierarchical system of crisis management in which national authorities take all relevant decisions even when they deploy localized strategies. There is no evidence suggesting that this model has been effectively challenged by sub-national actors or substantially reshaped, even when stretched and under pressure, during the first year of the pandemic.

### Italy and Spain: Attempts at Centralization in Competitive Regionalized Systems

When the pandemic started, Italy and Spain relied on a much more decentralized system of governance than France, particularly in the health sector. In fact, in both countries, healthcare is a core policy responsibility of regional governments and this even led to the emergence of divergent regional “welfare regimes” and fragmentation of social policies (Toubeau and Vampa 2020; Vampa 2016). In both countries decentralization was achieved by increasing levels of regional “self-rule” (Hooghe, Marks, and Schakel 2010) and transferring significant policy-making authority to regional governments, while keeping fiscal authority fairly centralized. The strengthening of the regional level, however, was not accompanied by robust (formal or informal) mechanisms of territorial coordination. This was clearly highlighted in Brözel’s account of Spanish “federalism” characterized by “scarce constitutional provision for intergovernmental cooperation and vertical integration” (Börzel 2002, 93), and conflictual relations between regional and national authorities. Italy is also characterized by high levels of vertical and horizontal territorial “competition”—whereby individual or small groups of regions seek to gain the best possible deal with the government for themselves (Giovannini and Vampa 2020).

The policy dynamics emerging in the first phase of the crisis reflected some of the territorial tensions that pre-dated the pandemic. Central governments started taking some actions in late January and early February. For instance, on 31 January a state of emergency was declared by the Italian government. This was based on a statutory—rather than constitutional—provision: the Civil Protection Act 2018, which empowers the government to adopt “any necessary measure” within the limits of the “general principles of the legal system” (Palermo 2020). In Spain the central Ministry of Health activated the COVID-19 protocol in coordination with the regional Departments of Health (Kölling 2020). However, these initiatives did not really erode the autonomy of regions and, when the real emergency started, there was quite a lot of confusion about the allocation of responsibilities.

Since healthcare is one of their “core” policy responsibilities (Toubeau and Vampa 2020), regional governments in both countries immediately found themselves at the forefront of the response to the outbreak. Central governments initially acted cautiously and incrementally. As highlighted by Capano (2020, 328), “in the case of the COVID-19 pandemic, the regional institutional arrangements of the Italian state emerged as a key dimension affecting the nature of the government response” with “key activities … organised very differently from region to region.” In Spain, the first closures of schools and cultural institutions were decided in an uncoordinated manner by the regional governments in Madrid, Basque Country and Catalonia (Royo 2020). Only at a later stage did the central government take direct action and call for the closure measures to be applied to all regions (Robinet-Borgomani 2020a). School closures were also at the center of a heated debate between regional and central governments in Italy, with regional presidents accusing the Prime Minister of failing to provide clear guidelines applicable to the whole country (Rubino 2020).

Centralization was eventually regarded as the only solution in the face of growing uncertainty. The Italian central government first passed an emergency decree imposing a regional lockdown in Lombardy and surrounding areas on 8^th^ March, when the number of cases had already started growing exponentially. After only one day, this initiative was followed by another decree enforcing a national lockdown. These decisions were made “without truly consulting the regions” (Capano 2020, 336). Yet, the inherently conflictual characteristics of territorial institutional arrangements in Italy did not allow the central government to simply act unilaterally and impose its authority like we observed in the French case. In fact, after passing the decrees, ministers in Rome had to “[re-]negotiate everything with the regions, leading not only to very confused institutional communication but also to many decision-making mistakes in terms of content and timing” (Capano 2020, 336).

In Spain recentralization was even more radical than in Italy but, equally, it met resistance and did not reduce—but in some cases exacerbated—competitive territorial dynamics. When it became clear that the situation was rapidly deteriorating and territorial coordination was hampered by “vertical fragmentation of powers,” the central government emerged as a “focal point” in crisis management (León and Garmendia Madariaga 2020, 9). The socialist Prime Minister Pedro Sánchez announced the declaration of the “state of alarm” on 13^th^ March. This was a constitutional measure proposed by the national executive requiring only the approval by absolute majority of the Spanish Lower Chamber (*Congreso*). It involved the total centralization of all lockdown measures in the hands of four central Ministers: Health, Defence, Transports and Home Affairs (Grau-Creus and Sanjaume-Calvet 2020). These measures were not previously negotiated with the regional governments, but, instead, were *de facto* unilaterally imposed, thus undermining the regional competence on health matters (Angel and Linera 2020). However, this strengthening of central authority vis-à-vis regional authority was quite fragile and precarious. First of all, Sanchez’s government lacked a majority in the Spanish Congress of Deputies and had to rely on difficult cooperation with the opposition, including regionalist and pro-independence parties. Regions could continue to take measures as far as they did not collide with central decisions. In fact, there was still some regional diversity in approaches and regulations (Nogueira López and Doménech Pascual 2020). Regions continued to clash with the central government over the allocation of competences and the nature of the lockdown, with Catalonia demanding tougher measures and calling for the region to be closed off from the rest of Spain (Dombley 2020).

Clearly, pre-crisis “causal forces” affected the way the pandemic developed in the two countries. When the virus started spreading, Spanish politics found itself in a worrying spiral of polarization and fragmentation, both ideologically and territorially, due to the rise of strong secessionist tensions in Catalonia, but also in other territories (Rodon 2020; Sánchez 2020). Thus centralization was confronted with those dynamics of “competition,” which Börzel (2002) had already described almost twenty years before in her assessment of “conflictual” interactions between Spanish state and regions responding to Europeanization.

**Figure 2:**
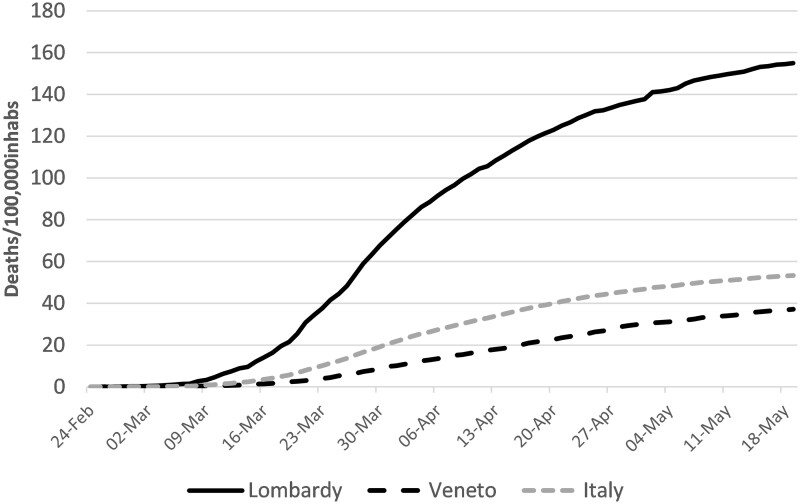
Deaths in Lombardy and Veneto (per 100,000 inhabitants) in the first three months of the pandemic.

In Italy regional politics was less confrontational than in Spain but still quite fragmented (Vampa 2015) and subject to centrifugal dynamics. This, combined with a regionalized health system, led to the development of different regional responses to the pandemic with diverging outcomes. This is well exemplified by the different paths followed by Lombardy and its neighbouring region, Veneto (Figure 2). Observers have pointed to the different health systems existing in the two regions and have shown that their respective leaders, despite belonging to the same party and being subject to the same national lockdown, did not converge in their policy responses (Giuffrida 2020; Johnson 2020). In sum, in a context of “weak centralization” and absence of coordination mechanisms favoring (good) “policy diffusion” (Vampa 2020) COVID-19 ended up reinforcing pre-existing territorial fragmentation.

In Spain, even though—not without difficulty and initial uncertainties—the central government managed to re-arrange “the decentralized health system into a *temporary* higher degree of centralization” during the emergency, the relaxation of lockdown measures occurred on a “case-by-case basis” (Henrìquez et al. 2020, 573, italics added). This also led to the re-emergence of territorial tensions, which worsened when the second wave of the pandemic started in summer and the absence of a consistent and cohesive territorial strategy became clear. For instance, between September and October, the Autonomous Community of Madrid repeatedly clashed with central government over the imposition of tougher restrictions while COVID-19 cases were rising at worrying rates (Sevillano and Viejo 2020). Additionally, the emergency does not seem to have weakened centrifugal political pressures, as clearly shown by the increased majority won by pro-independence parties during the Catalan regional elections in February 2021 (Cetrà and Keating 2021). In sum, territorial policy dynamics have not shifted toward a new institutional equilibrium of increased cooperation and coordination. Rather, after an abrupt recentralization of policies in the face of an external shock, the issue of how authority should be distributed across different levels of government remains the object of a heated and unresolved political debate.

### Germany: Building Consensus and Coordination

Centralizing pressures were not absent in Germany, which, despite being a federal republic, also has mechanisms that transfer substantial powers to Berlin in the event of a major crisis like COVID-19 (Siewert et al. 2020, 1). Since disease control falls within the competence of the *Länder*, the central government chose to rely on a federal statute called the *Infektionsschutzgesetz* (Infection Protection Act, IfSG), in order to strengthen its position vis-à-vis sub-state authorities (Dostal 2020, 548; Saurer 2020). Yet, unlike the cases described above, central government avoided acting unilaterally and did not attempt to bypass regional actors. Rather, it played the role of coordinator and mediator among the 16 *Länder.* This was particularly important because, even though the IfSG gave the Federal Health Minister greater power and the chance to use this power without consultation with the cabinet (Siewert et al. 2020, 3), ultimately it entrusted regional governments—in federal Germany these are “state” governments—with implementation of the legislation. This was in line with the cooperative character of German federalism, where legislation is concentrated at the federal level, while execution and administration are largely delegated to the *Länder* (Börzel 2002, 46). Regional governments accept to execute central legislation because they can affect its content by participating in decision-making processes with the federal government not only “officially” via the *Bundesrat—*the legislative upper chamber—but also in a number of less constitutionally embedded (but still quite institutionalized) intergovernmental forums. More generally, also in a situation of extreme emergency like COVID-19, dynamics of “compensation-through-participation” (Börzel 2002, 46) allowed regional governments to accept a certain loss of “self-determination” in exchange for some level of co-decision in the national response to the pandemic.

Of course, there were initial differences in regional approaches and tensions between central and regional governments and also among regional governments. Additionally, the *Länder* retained a significant level of “decision-making capacity,” creating potential for an “uneven landscape of policy actions,” which, however, had a “minimal” impact on “immediate health outcomes” (Desson et al. 2020, 410). What prevented Germany from experiencing substantial divergence in policies and outcomes was a set of “existing coordinative mechanisms” (Schnabel and Hegele 2020). By holding meetings on a regular basis, the Chancellor and regional leaders were able to ensure a relatively uniform approach to crisis management. Following a truly multi-level logic, the decisions reached at the meetings were implemented by the *Länder* through executive orders and administrative regulations that the local authorities implemented (Schnabel and Hegele 2020). Thus, as described by Siewert et al. (2020, 7),

the 16 *Länder* governments … and the federal government quickly converged on common strategies for controlling the spread of the virus. To “flatten the curve,” the states initially all copied virtually every restrictive measure adopted by any one of them within a few days.

So “harmonization” was not centrally imposed but emerged gradually through coordinated interactions. These occurred both vertically—between national and regional governments—and horizontally—among regional governments––, thus also favoring policy diffusion. Once again, Börzel (2002) effectively showed that this mix of vertical and horizontal dynamics had been in place well before the pandemic started and had helped reducing tensions associated with centralizing tendencies in previous phases of German recent history. As highlighted by Kuhlmann et al. (2021, 15), the initial response to COVID-19 followed a “bottom-up” logic of decentralized governance leading to coordination, which reflected Germany’s tradition of “unitary” federalism. So the system did not have to be created from scratch, and this makes the German case quite different from the Spanish and Italian ones, where decision-making forums, involving central government and regions, were more *ad hoc* and much less institutionalized.

Besides institutional legacies, other political factors contributed to fostering cohesiveness. Despite the increasing fragmentation of the German party system (Bräuninger et al. 2019), centrifugal territorial tendencies were limited by a long-standing tradition of coalition and consensual politics. The parties in central government—Christian Democrats (CDU/CSU) and Social Democrats (SPD)—were also included in all 16 German *Länder*, either forming grand-coalitions similar to the one in Berlin or building alliances with other parties. Unlike Italy and Spain, where regional politics has significantly diverged from national one, thus becoming more territorially fragmented, in Germany the emergence of “region-specific” political actors has been a rare phenomenon. Sub-national politics is still dominated by national parties.

Bavaria can be regarded as a partial exception, since, for decades, it has been ruled by a regionalist party, the Christian Social Union in Bavaria (CSU). This party has been traditionally supportive of more autonomy and Bavarian exceptionalism, while, at the same time, being a key actor in central government (Vampa and Scantamburlo 2020). It is therefore not surprising that Bavaria, which was initially more affected by the virus than other areas of the country, was the only *Land* that declared a state of emergency during the first wave (Siewert et al. 2020, 6). Its Minister-President, Marcus Söder (CSU), was also the first advocating and introducing stricter measures than those decided at the federal level (Robinet-Borgomani 2020b). Yet other states followed suit (Milbradt 2020) and convergence dominated the first phase of the emergency. Additionally, rather than playing the role of the “outsider” in national debates, Söder gained significant national support, becoming the second most popular politician after Angela Merkel.^1^ Again, following a bottom-up logic, the regional arena contributed to shaping national politics, rather than fracturing it.

Of course, tensions re-emerged after the peak of the pandemic, with *Länder—*including initially cautious Bavaria—breaking ranks with Berlin and supporting a quick reopening of the economy (Chazan 2020). Yet this did not result in dramatic breakups. Tensions could still be managed within the structure of a federation that had proved capable of combining the benefits of a decentralized approach—particularly in the case of testing (Oltermann 2020)—with high levels of cohesiveness and coordination. The second and third waves between winter 2020 and spring 2021, which saw the number of infections and deaths increase significantly, did not seem to fundamentally undermine this model of crisis management. At the same time, the problems experienced by Germany at the start of its vaccination rollout determined a shift in public perceptions: the positives of its coordinated and cooperative federalism were quickly converted into negatives (Oltermann 2021). It remains to be seen how resilient and adaptable German institutions will be as the crisis evolves and new challenges (and opportunities) emerge.

### The United Kingdom: A “hybrid” System in Search of a Consistent Strategy

Among the five cases considered here, the United Kingdom is perhaps the most complex and difficult to categorize. Many observers have focused on the intergovernmental relations between England, Scotland, Wales and Northern Ireland before and after the pandemic (McEwen et al. 2020). Much less attention has been paid to what occurred *within* England, where almost 85 percent of the British population lives. If we look at the interactions between central government and devolved nations, we can identify some of the dynamics discussed in the previous sections. They oscillated between competition, following a pattern similar to the one observed in Italy and Spain, and coordination, more similar to the German model. Yet what makes the United Kingdom a truly “hybrid” system is the co-existence of substantial policy making-autonomy in the devolved administrations and over-centralization in England. Given its size, England cannot just be considered as a “region” of the United Kingdom. Rather, it is a nation without “meso-level” government structures comparable to those of Italy, Spain, Germany and even France. Lack of consistency in territorial governance is also determined by the “dual-hatted” role played by the UK government, which “claims to speak for the entirety of the UK while also, simultaneously, being required to speak for England as a territorial entity” (McEwen et al. 2020, 638).

Therefore, it is not surprising that confusion dominated the initial phase of the pandemic. There were problems in communicating with the public about whether certain policies and announcements applied to the United Kingdom as a whole or only to England. Testing was an example of this ambiguity, with the Health Secretary promising that 100,000 COVID-19 tests would be carried out every day by the end of April. There were mixed messages about whether this pledge was in fact for England only, and the Scottish and Welsh governments subsequently announced their own plans for scaling up testing (Paun, Sargeant and Nice 2020, 6). Generally, COVID-19 arrived at a time of “constitutional flux” (Mullay 2020), with the UK’s decision to leave the EU (Brexit), placing “a huge strain” on intergovernmental relations (McEwen et al. 2020, 632). Sharp disagreements had emerged between the devolved governments on the one hand, and the UK/English government on the other, during the process of legislating for the UK’s EU exit, thus exposing the flaws in a territorial system based on asymmetrical devolution arrangements and lacking structures of cooperative government (McEwen et al. 2020, 634).

Yet, surprisingly, the coronavirus crisis also incentivized coordination between the four nations. This became apparent when, in early March, the UK and devolved governments published a joint Coronavirus Action plan, “a rare sighting of government policy paper that was co-branded by the four administrations” (Paun 2020). Additionally, they worked together on the Coronavirus Act, which was eventually passed in Parliament and conferred powers on all four UK governments to tackle the pandemic. This clearly contrasted with the devolved governments’ refusal to support Brexit legislation only a few weeks before (Constitutional Unit 2020). Leaders of the devolved administrations participated in meetings of the so-called “COBRA” emergency committee throughout this period, helping to ensure that major announcements, including the imposition of the lockdown in late March, were coordinated between the capitals (Paun 2020). The relatively quick shift from harsh territorial competition during the Brexit process to a more coordinated approach at the start of the pandemic was a development highlighted by a number of observers (see, for instance, Gallagher 2020; Paun 2020). Grace (2020) argued that the pandemic “had a unifying effect … as a function of … the essentials of coordination and collaboration needed across all parts of the UK geography and polity.” The shift was also facilitated by the flexible and uncodified nature of the UK unwritten constitution (Young 2018, 17), which allowed a rapid change in intergovernmental relations as soon as key actors in central and devolved governments showed a shared willingness to pool responsibility in the face of an unprecedented crisis. The fact that only four territorial units were involved in the process may have also favored rapid convergence around a “benchmark” (Weaver 2020, 174), once political conditions changed.

Yet this did not lead to a new (lasting) “equilibrium” based on a significantly more stable and “institutionalized” system of intergovernmental coordination and cooperation than before the crisis. Communication about the parts of the country to which certain policy decisions applied remained quite ambiguous (Paun, Sargeant and Nice 2020, 6). Additionally, the quick move to cooperation could be easily reversed if key actors’ preferences changed in opposite directions—something quite likely to occur, since each nation of the Union was governed by a different party. As the crisis unfolded, divisions between devolved and central governments soon re-emerged. After the pandemic reached its first peak, the four nations clearly started diverging and adopted different “exit strategies” (Fairgrieve 2020). While England moved from a “stay at home” to a less strict “stay alert” policy, the leaders of Wales, Scotland and Northern Ireland kept sticking to the latter and openly criticized what was being decided in London (Mason and Siddique 2020; McCaffre 2020). Moreover, the Scottish Government’s more cautious and measured approach to tackling the pandemic and its ability to “outperform”—at least in terms of communication—the central government, soon had a positive impact on support for Scottish independence (Parker 2020). In sum, far from leading to lasting convergence and coordination, COVID-19 amplified pre-existing contradictions and tensions in the British territorial settlement.

However, looking at the interactions between the four UK nations tells us only part of the story. As mentioned above, the overwhelming majority of the British population experienced the first wave of the pandemic in a highly centralized political system: England. It is what happened within England that made a decisive contribution to the overall performance of the United Kingdom. Giovannini (2020, 40) has effectively highlighted that in England, “Westminster … almost instinctively entered in ‘top-down command and control’ mode—centralising even further decision-making.” At the same time central responses to the crisis were “poor and often contradictory” with “decisions imposed by the centre on local authorities,” which had negative effects on local communities:

from the appalling management and distribution of Personal Protective Equipment and continued delays in the sharing of data on infection rates, to the quickly-withdrawn promise made to local authorities to “spend whatever it takes” to respond to the pandemic (Giovannini 2020, 40).

The absence of an English devolution agenda characterized by “any meaningful, subsidiarity-informed democratic settlement” had been acknowledged well before the coronavirus crisis (Richard and Smith 2015, 385). Hambleton (2017) showed the limitations of the modest attempts at transferring power to sub-national authorities made over the last decade. Like regionalization in France (see above), devolution was a misused term to define a process of “super-centralization” in which central authorities determine key policy targets and allocation of (vanishingly small) funds (Hambleton 2017, 7). It is therefore not surprising that during the pandemic, representatives of English “combined authorities,” like the mayor of Greater Manchester, Andy Burnham, criticized the government’s “London-centric” approach (Williams and Britton 2020) and observers questioned the ability of a highly centralized system to cope with the emergency (Watts 2020). To be sure, the absence of strong regions developing different health policies might explain why the initial impact of the pandemic in the United Kingdom, of which England is by far the largest constituent unit, was not as territorially differentiated as in Italy and Spain. This, however, did not prevent the United Kingdom from becoming the country with the worst death toll in Europe at the end of the second wave. The lack of powerful “meso-level” authorities also meant that, in large part of the British territory, the negative effects of wrong central government’s decisions were not “moderated” by any significant sub-national resistance.

The introduction of local lockdowns first and a “tier system” within England in the autumn of 2020 did not lead to a more decentralized approach (Iacobucci 2020). In fact, the system, enforcing different restrictions depending on the alert level in English local authorities, was designed by central government without much consultation with other regional actors. Rather than leading to a more bottom-up management of the crisis, it eventually resulted in a “centralised but fragmented system with little incentive to co-operate” (Thomas 2020). In summer the first local lockdown in Leicester (imposed by central government) was dominated by a dispute over the lack of powers and data available to local authorities. In autumn, after the introduction of the tier system, multi-level tensions reached a new high during Manchester’s move into tier 3 lockdown control (the highest one), with its Mayor refusing to implement the government’s strategy due to the lack of financial support (Walker et al. 2020).

In sum, the later phases of the pandemic in 2020 saw a reassertion of centralism in England, which, differently from what had happened during the first wave, triggered more confrontational local reactions. At the same time, the establishment of a centralized vaccination system in January 2021 and the rapid rollout of jabs in the first trimester of the year contributed to re-establishing a certain level of confidence in the ability of national authorities to respond effectively to the emergency.

## Discussion and Conclusion

The analysis of the five cases presented above shows that there was significant variation in the territorial policy dynamics triggered by the pandemic. Although COVID-19 resulted in increasing activism of central governments in all five countries, it also led to very different patterns of central-regional interactions. Table 1 summarizes the key features of each case. It is shown that the impact of COVID-19 on policy dynamics was moderated by pre-crisis “causal forces,” particularly by institutional and political factors, which pre-dated the pandemic. In France the crisis reinforced centralization trends and the hierarchical character of territorial governance. In Italy and Spain attempts at centralization were met with increasing resistance. Central institutions struggled to harmonize health systems and models of governance, which had diverged for years before the pandemic. The territorially fragmented character of party competition in the two countries—especially in Spain—also hindered the establishment of a hierarchical system similar to the one existing in France. It also prevented the emergence of cooperative territorial interactions as stable as the ones observed in Germany, where a more cohesive political system and a long tradition of “compensation-through-participation” allowed central government to act as a coordinator and mediator between diverging positions of the *Länder* and promote convergence. The United Kingdom appears to be the most ambiguous case, combining instances of coordination and competition between the four nations of the Union, while relying on a highly centralized system in England, where more than 80 percent of the British population lives. Again, pre-crisis factors explain the “hybrid” character of policy dynamics emerging in response to the pandemic. In particular, the process of devolution started in the 1990s did not contribute to the establishment of stable mechanisms of intergovernmental cooperation/coordination among the four nations (which also diverged in terms of political leadership). At the same time, the devolution agenda in England remained a top-down process, which many observers even regarded as an attempt to promote further centralization. Coronavirus interacted with all these trends in the United Kingdom and gave rise to the inconsistent responses described above.

**Table 1.** The territorial impact of COVID-19 in five European countries

Country	Pre-crisis causal forces	Territorial policy dynamics
France	Regionalization reforms strengthening the role of central government (reduced number of regions); prominence of national politicians/national officials	Hierarchical
Italy	Healthcare as “core policy responsibility” of regions; high regional policy making autonomy, low fiscal autonomy; asymmetric decentralization; territorially fragmented party systems; regionalist parties	Competitive
Spain	Healthcare as “core policy responsibility” of regions; high regional policy making autonomy, low fiscal autonomy; asymmetric decentralization; territorially fragmented party systems; strong regionalist and secessionist parties	Competitive
Germany	Regions enjoy high “decision-making capacity” and autonomy in implementing central legislation; *Länder* involved in national decision making via *Bundersat* and other more informal, but highly institutionalized, forums; territorially integrated party system and tradition of “consensus” politics (despite recent challenges)	Cooperative/Coordinated
United Kingdom	Asymmetric devolution arrangements; England by far the largest unit (including 85% of the population) and highly centralized; intergovernmental relations only involve four units but no established mechanisms of coordination comparable to German ones; each nation of the union is ruled by a different party	Mixed (prevalence of hierarchical dynamics in England)

Of course, each case did not perfectly correspond to an “ideal-type.” Germany was also characterized by competitive pressures and one may argue that, in fact, there were some territorial differences in the impact of the virus between, say, Bavaria and Brandenburg. Yet the comparative approach adopted in this article places country-specific assessments of the crisis in a broader perspective. What may look as “extreme” policy fragmentation to a German observer focusing on her own country, is in fact minimal divergence when compared to what happened in other countries. The same can be said, for instance, when discussing strong government intervention in Italy. An Italian observer may regard the actions taken by Rome as a clear sign that the country shifted toward a strictly centralized and hierarchical system of governance. Yet a comparative assessment clearly shows that, in the Italian case, centralization was much more moderate and affected by regional factors than in France.

The analysis presented here has mainly focused on the responses to the pandemic until the beginning of 2021. At the time of writing (April 2021), the crisis is still ongoing and we have entered a new era, in which vaccines against COVID-19 are available. Since the start of the vaccination campaign, the United Kingdom, which relies on a highly centralized system managed by its National Health Service (NHS), seems to have significantly improved its position after initially being one of the worst affected countries in Europe and the world. It remains to be seen whether a new system of territorial governance will emerge as a result of this change in circumstances.

Clearly, the policy dynamics described here may change over time. This is also due to the evolving nature of the crisis, which has moved from the need to develop a defensive strategy aimed at limiting the spread of the virus (in 2020) to actively building capacity for mass immunization (in 2021). The framework and analysis presented in this contribution are certainly open to refinements and additional evidence, and may be used as a starting point for future studies. The latter should provide more long-term assessments of how multi-level systems adjust to the spread of a virus that has strained societies, economies, and polities across the globe.
